# Liquid chromatography-mass spectrometry platform for both small neurotransmitters and neuropeptides in blood, with automatic and robust solid phase extraction

**DOI:** 10.1038/srep09308

**Published:** 2015-03-20

**Authors:** Elin Johnsen, Siri Leknes, Steven Ray Wilson, Elsa Lundanes

**Affiliations:** 1 Department of Chemistry, University of Oslo, PO Box 1033, Blindern, NO-0315, Oslo, Norway; 2 Department of Psychology, University of Oslo, PO Box 1094, Blindern, NO-0317, Oslo, Norway; 3 The Intervention Centre, Oslo University Hospital, PO Box 4950 Nydalen, NO-0424 Oslo, Norway

## Abstract

Neurons communicate via chemical signals called neurotransmitters (NTs). The numerous identified NTs can have very different physiochemical properties (solubility, charge, size etc.), so quantification of the various NT classes traditionally requires several analytical platforms/methodologies. We here report that a diverse range of NTs, e.g. peptides oxytocin and vasopressin, monoamines adrenaline and serotonin, and amino acid GABA, can be simultaneously identified/measured in small samples, using an analytical platform based on liquid chromatography and high-resolution mass spectrometry (LC-MS). The automated platform is cost-efficient as manual sample preparation steps and one-time-use equipment are kept to a minimum. Zwitter-ionic HILIC stationary phases were used for both on-line solid phase extraction (SPE) and liquid chromatography (capillary format, cLC). This approach enabled compounds from all NT classes to elute in small volumes producing sharp and symmetric signals, and allowing precise quantifications of small samples, demonstrated with whole blood (100 microliters per sample). An additional robustness-enhancing feature is automatic filtration/filter back-flushing (AFFL), allowing hundreds of samples to be analyzed without any parts needing replacement. The platform can be installed by simple modification of a conventional LC-MS system.

NTs are metabolites used for communication between neurons in the brain. In humans, analysis of cerebrospinal fluid (CSF) gives the most direct measure of central NT levels. Due to their less invasive nature, neuroimaging approaches such as positron emission tomography (PET) and magnetic resonance (MR) spectroscopy^1^,^2^ are promising alternatives. However, the cost, availability and practicality aspects of CSF extraction and neuroimaging have led many studies to rely on quantification of NTs from blood, saliva or other peripheral fluids.

Measuring levels of various NTs (e.g. GABA, serotonin and its precursor tryptophan, adrenaline, oxytocin and vasopressin) in blood or other bio-fluids gives valuable information for several purposes. Some studies point to differences in peripheral NT levels as potential biomarkers for psychiatric conditions (e.g. serotonin^3^), or use blood measures of NTs to validate experimental neuroscience methods^4^,^5^. Peripheral NT levels have also repeatedly been shown to correlate with e.g. parent-infant attachment, e.g. neuropeptides oxytocin and vasopressin^6^. Central oxytocin (OT) levels are thought to mediate the link between OT levels in plasma and behaviour, although the highly limited ability of many NTs to cross the blood brain barrier render the relationship between central and peripheral levels less clear^7^. Therefore, reliable, valid and sensitive analytical methodologies are required to further establish the roles and predictive traits of peripheral NTs.

A further complication of employing peripheral NT levels is that reliability and validity can be questionable. Different methods of quantification do not always give comparable results. For OT, McCullough *et al*^8^ recently pointed to a discrepancy of up to 100-fold between conventional radioimmunoassay (RIA) and the newer, commercially available enzyme immunoassay (EIA) approach. The authors suggest that such EIA measures may misattribute other molecules to OT. Moreover, RIA and EIA methodologies are intended for specific compounds, and can be very time-consuming to establish, reducing method flexibility for e.g. identifying novel metabolite candidates for brain/blood level correlations^9^.

Another obstacle is the difficulty with obtaining convincing data from small amounts of sample. Recently, Clark *et al*^10^ analysed cerebrospinal fluid (CSF) samples from human infants and showed a correlation between neuropeptide levels and behaviour. The authors were however unable to address the correspondence between plasma and CSF peptide levels, since the amount of blood required for conventional analysis of plasma OT exceeded the amount that could safely be drawn from neonates.

An alternative to RIA and EIA is mass spectrometry (MS) based methodology. MS provides detailed information of detected compounds, including molar mass, charge, and (clues to) its molecular structure (when employing tandem MS). MS is commonly coupled with liquid chromatography (LC), which separates compounds in a sample from each other, prior to MS detection. This step enhances e.g. sensitivity, as fewer compounds enter the MS per time, reducing ion suppression^11^. LC-MS is common instrumentation in modern analytical laboratories, and is associated with excellent validity and high accuracy/precision measurements, especially when internal standards are used, e.g. adding known amounts of an isotopically labelled version of each analyte (e.g. the NTs) to the sample, which are used to correct for variances that can occur during sample preparation/LC-MS recording.

However, reported LC-MS methods for NT determinations are often intended for rather large amounts of sample^12^,^13^,^14^ and may therefore not be suitable for analysis of limited, precious samples from e.g. infants and blood banks. Also, they are typically not suited for measuring all classes of NTs at once, due to the significant variation of physiochemical properties of NTs, such as hydrophobicity and charge. Simultaneous determination of members from all NT classes would be desirable as NTs can interact extensively, as is well documented across human and non-human models. LC-MS methods may however also require a number of manual preparation steps, e.g. enriching the compounds with solid phase extraction (SPE) columns prior to LC-MS, thereby increasing analysis cost.

Our goal was to develop an LC-MS platform that would be capable of analysing samples to quantify all NT classes. Moreover, we wanted the platform to have high sensitivity, allowing analysis of minute samples. In addition, we wanted the platform to be automated, but at the same time very robust. Our proposed solution is to employ LC-MS, with LC performed in hydrophilic interaction mode (HILIC)^15^, coupled with on-line solid phase extraction (SPE)^16^ and automated filtration/filter backflushing (AFFL)^17^. The LC column was in capillary format (cLC). The SPE step is also performed in HILIC mode, and is compatible with protein precipitation using acetonitrile (ACN) with 0.1% formic acid (FA), allowing direct analysis of the supernatant with no additional steps. We will here describe the versatility of the method and demonstrate its sensitivity, quantification, reliability and other traits.

## Results

**Figure 1:**
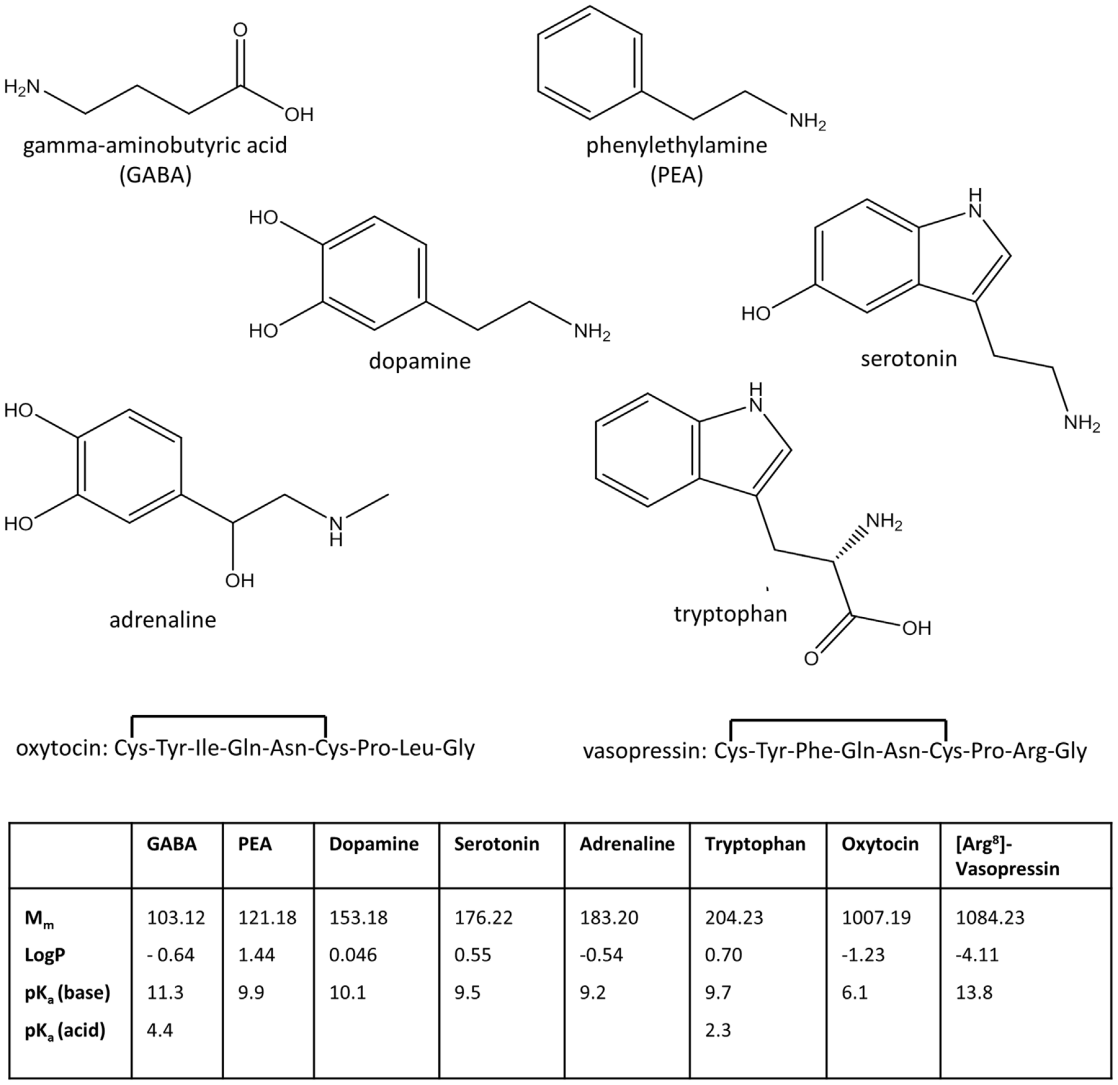
Neurotransmitter molecular structures and info. Molecular structures of the six small neurotransmitters and the amino acid sequence of the two neuropeptides. A table with molar mass (M_m_), octanol-water partition coefficient (log P) and pK_a_ values of all analytes are included (values obtained from SciFinder (CAS, Columbus, OH, USA)).

**Figure 2:**
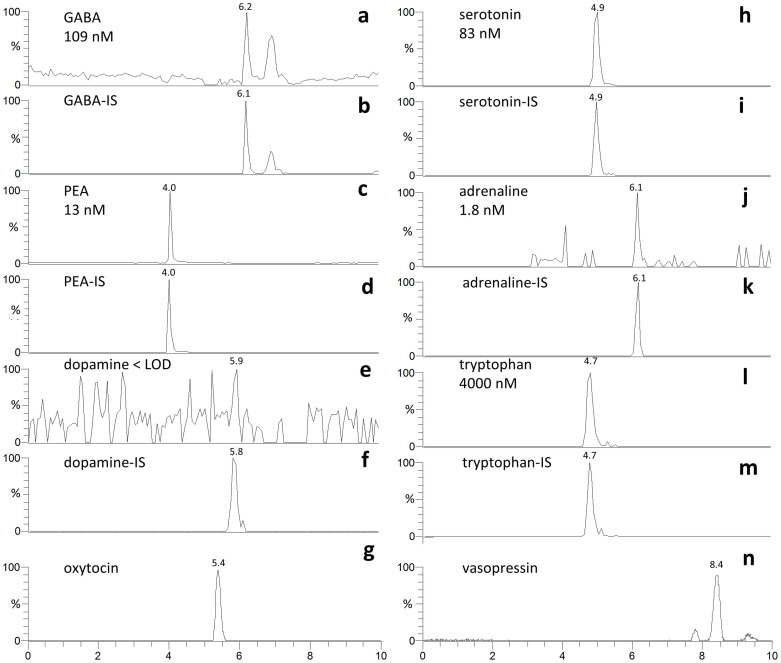
LC-MS chromatograms of neurotransmitters identified in whole blood sample. Chromatograms showing the endogenous NTs identified/measured in a whole blood sample from a healthy volunteer with no medical history of importance. The endogenous NT peak is compared with the internal standard (IS) peak and the retention times are included. (a) GABA (plate number N = 16 000), (b) GABA-IS, (c) PEA (N = 12 000), (d) PEA-IS, (e) dopamine (no N calculated), (f) dopamine-IS, (g) oxytocin (N = 6000, no IS available), (h) serotonin (N = 6000), (i) serotonin-IS, (j) adrenaline (N = 19 000), (k) adrenaline-IS, (l) tryptophan (N = 4000), (m) tryptophan-IS and (n) vasopressin (N = 10 000, no IS available). All chromatograms were obtained from the same recording.

**Figure 3:**
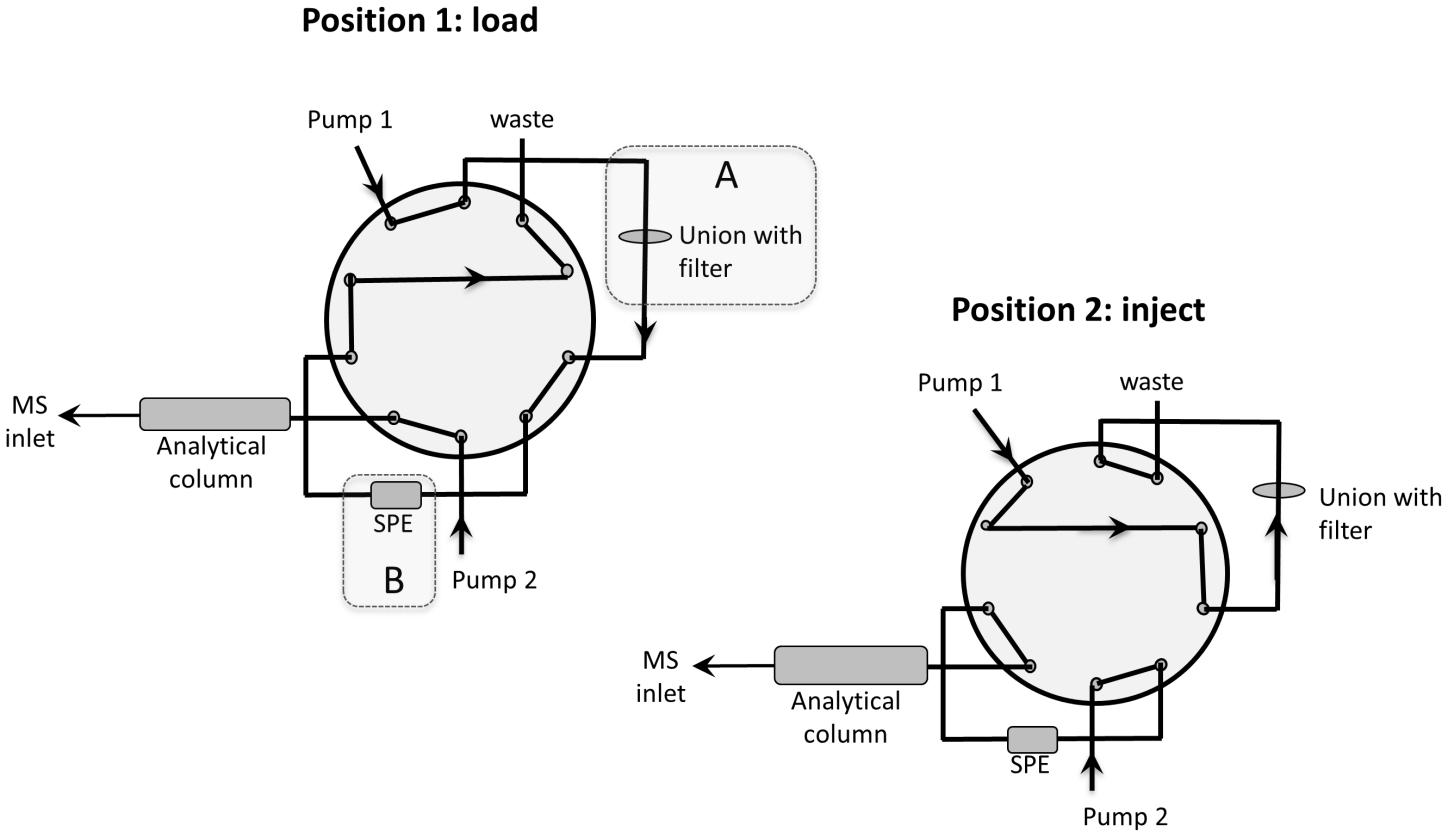
Illustration of AFFL-SPE-cLC-MS system. The two switching positions of the AFFL-SPE system, (1) “load” and (2) “inject” are illustrated. The shadowed areas A and B highlight the AFFL filter and the SPE, respectively.

**Figure 4:**
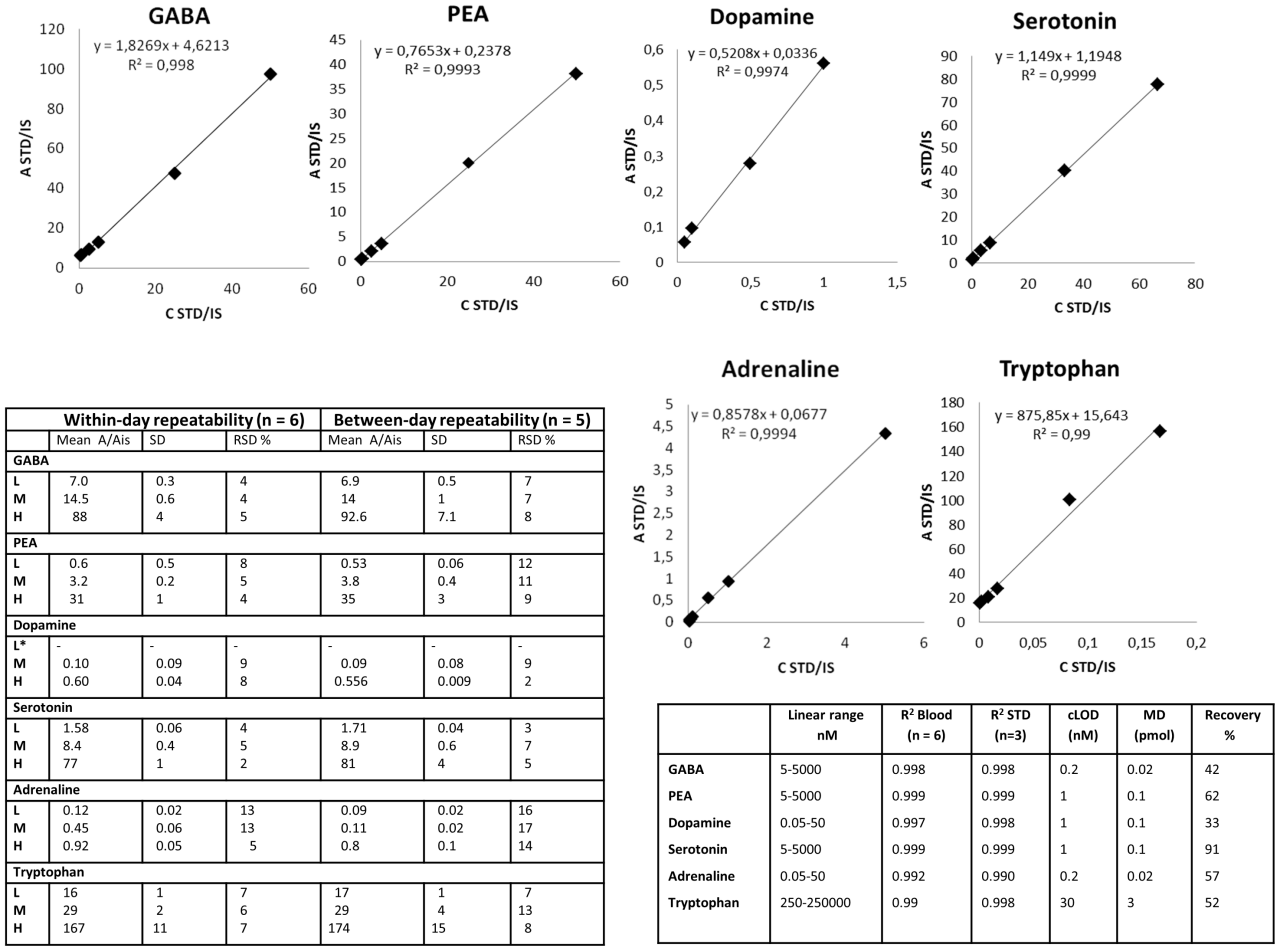
Validation data. Calibration curves for six NTs in spiked whole blood, including regression equations and R^2^ values (n = 2 for each level). The non-zero values for b in “y = ax + b” are due to endogenous levels of the neurotransmitters in blood. The table to the right show validation data including linear range, R^2^ values (in blood and standards), concentration limits of detection (cLOD), minimum detectable amounts (MDs) and recovery (%). The table to the left shows the within-day and between-day repeatabilities of the NTs in spiked whole blood at the concentration levels L (low), M (medium) and H (high). * No result for dopamine was obtained at the concentration level L, due to s/n < 10.

The main results were: neurotransmitters/precursors with a wide range of physiochemical properties (see structures and other data, Fig. 1) could be identified/measured in a single analysis, needing only 100 μL of whole blood (see cLC-MS chromatograms of endogenous NTs and isotopically labelled internal standards (IS), Fig. 2). Identifications were based on retention time matching with standards and spectra obtained with high resolution MS. A novel, automated AFFL-SPE-cLC-MS analytical platform was developed for this purpose (Fig. 3 and Supplementary Fig. S1). The automated platform could be used for reliable trace determinations, evaluated with NT monoamines and amino acids with readily available labelled internal standards; see calibration curves and validation tables (Fig. 4). Due to the platform's sensitivity and versatility, trace levels of the much larger neuropeptides oxytocin and vasopressin could also be easily detected (Fig. 2g and n). The platform provided good and similar chromatographic quality for monoamines, amino acids and peptides (Fig. 2), suggesting that AFFL-SPE-cLC-MS is a suitable platform for comprehensive NT determinations in small samples. Below is a more detailed presentation of the results and technical aspects.

### Method development

Initially, two commercial zwitter-ionic HILIC columns for cLC separations were examined, (a) ZIC-HILIC^18^ and (b) ZIC-cHILIC^19^. Both have zwitter-ionic stationary phases, but while (a) has a sulphobetaine type functionality (quaternary ammonium part = positive charge, sulphonic acid part = negative charge), where the negative charge is the most accessible to interacting solutes, (b) has a phosphorylcholine type functionality (phosphate part = negative charge, quaternary ammonium part = positive charge), where the positive charge is the most accessible. Neither of the two 15 cm long columns provided a baseline separation of all the NT analytes. However, since the analytes have distinct retention times, exact masses and/or MS/MS transitions (Table 1) and the platform provides satisfactory quantitative performance (see below), baseline separation was not considered to be a necessity. The efficiency (plate number N, see Supplementary note Calculation of efficiency), and asymmetry factor (A_s_, see Supplementary Fig. S2, for details) of the platform were somewhat better with (b) (see N values in caption of Fig. 2), hence the ZIC-cHILIC column was employed. Best resolution (R_s_) and N were achieved with a mobile phase consisting of 70% ACN and 30% 100 mM ammonium formate at pH 3 (see Supplementary note Mobile phases, for brief discussion on other mobile phases examined). In addition, 300 μM ascorbic acid (anti-oxidant) was added to the mobile phase to avoid online-oxidation effects^20^,^21^ (see Supplementary note Online oxidation).

An AFFL set-up^22^ was used for on-line sample filtration (Fig. 3, part A), using a stainless steel filter, and subsequent back-flushing of the filter. This approach eliminated the need for single-use filters, and manual handling of these, thereby allowing for hundreds of whole blood sample injections without hardware replacement (including filter, columns etc.). On-line SPE^16^ (Fig. 3, part B) was employed to automatically enrich the NTs prior to cLC separation, eliminating also the need for single-use SPE cartridges (and manual handling of these). Best performance was obtained using a ZIC-HILIC SPE column, compared to porous graphitic carbon- and ion exchange SPEs (see Supplementary note SPE column materials, and discussion below), with a 100% ACN loading solvent and a two minute SPE loading time. A ZIC-cHILIC SPE column was not available to us during the study, and therefore not investigated. At least 100 μL of acetonitrilic sample volumes (resulting from the protein precipitation step, see Methods section below) could be loaded on to the SPE without an analyte breakthrough occurring (see Supplementary Fig. S3). The cLC mobile phase successfully eluted the NT analytes from the SPE to the cLC column, and the analytes were chromatographed/detected in less than 9 minutes. Including the two-minutes loading time and one-minute post-run SPE re-equilibration time, the platform analysis time cycle was 12 minutes per sample. The only sample preparation was a one-step ACN (with 0.1% FA) precipitation.

### Method validation

Viewing Fig. 4 in more detail, the within-day and between-day repeatabilities (n = 6 and n = 5, respectively) were satisfactory for quantification, i.e. ≤20% relative standard deviation (RSD), as according to Federal Drug Agency (FDA) validation guidelines^23^, from lowest concentrations (between 0.05 nM and 250 nM, depending on the analytes expected endogenous levels) and up to three orders of magnitude, with good linearity (r^2^ = 0.990 to 0.999) in both spiked blood and aqueous standard samples using the Orbitrap MS. Of the analytes investigated, serotonin had the lowest RSDs (within-day: 2–5%, between day: 3–7%) while adrenaline had the highest RSDs (within-day: 5–13%, between-day: 14–17%). In blood, the recoveries of the (spiked) neurotransmitters varied from 33% for dopamine up to 91% for serotonin, which were corrected for using isotope labelled internal standards added prior to sample preparation, which was one-step protein precipitation with ACN and 0.1% FA, see Methods section below). Oxytocin and vasopressin (endogenously present at low pg/mL levels^8^) were also easily detected at quantifiable levels (i.e. signal to noise (S/N) > 20) with the same sample preparation. The concentration limit of detection (cLOD) varied from 0.2 nM for GABA and adrenaline, up to 30 nM for tryptophan (see Supplementary note Calculation of cLOD). For all analytes, carry-over was below the LOD (S/N < 3) for all concentrations tested, except the “XH” level (see Supplementary Table S4 for details), which was approximately 50 times higher of that expected of endogenous levels. Hence, blank injections between samples were not necessary. The same columns (SPE and analytical) were used for the entire study (>1000 injections) without need for regeneration/cleaning.

The analyte stability in whole blood was demonstrated by re-analysing a number of compounds in samples after 1 week storage at 4°C and −20°C, after 24 h, 48 h and 1 week at room temperature, and after two freeze/thaw cycles. The analyte peak areas from the re-analyses were compared to the initial peak areas from day 1. At 4°C, −20°C, and after 2 freeze-thaw cycles, the stability was satisfactory for all the neurotransmitters in the sample (i.e. no decrease in peak areas). Serotonin and GABA were stable at room temperature for 48 h, but not 1 week, while the dopamine peak area was somewhat reduced after only 24 h at room temperature. The other neurotransmitters were stable at room temperature for at least 1 week. In aqueous standards (diluted with ACN/H_2_O, 70/30), some degree of instability was observed at room temperature and 4°C over night, hence fresh working solutions were made daily, and the stock solutions were stored at −80°C.

## Discussion

Neurotransmitters/precursors could be identified/measured in a single analysis, needing only 100 μL of whole blood, using a novel automated AFFL-SPE-cLC-MS analytical platform.

Several LC-MS methods^12^,^13^,^14^,^24^,^25^,^26^ have been reported for polar NT determination, but disadvantages can be the need for extensive manual sample preparation and large sample amounts, etc. For the more hydrophobic neuropeptides, standard reversed phase (RP) stationary phases have traditionally been used^27^,^28^. Disadvantages of such approaches are that they are limited to compounds of some hydrophobicity, and can therefore not be applied for small polar NT without the use of ion-pairing reagents (typically not compatible with MS) or chemical derivatization of the analytes (significantly adding to time spent on sample preparation). To our knowledge, no previous reports have demonstrated the ability to simultaneously handle both very hydrophilic and hydrophobic NTs.

Hence, key advantages of the presented AFFL-SPE-cLC-MS platform include simultaneous handling of polar NTs as well as neuropeptides with enhanced sensitivity (enabling analysis of small samples), a high degree of automation and increased speed.

The sensitivity is much attributed to the high-end mass Q-Exactive™ Orbitrap spectrometer^29^ employed, but also to the use of capillary columns; using cLC reduces radial dilution of the analytes during chromatography compared to conventional columns^16^, and employs lowered flow rates; both these features are associated with elevated sensitivity in ESI-MS^16^,^30^. The sensitivity of the platform allowed for analyte determinations with internal standards or identification of all compounds except for dopamine, which was present below LOD (S/N ≤ 3). This was somewhat expected due to its rapid metabolism. Therefore, its lack of signal served *de facto* as a negative control, but will likely be measured in other samples, as the cLOD (determined with spiked samples) was relatively low (below 1 nM). Conventional bore HILIC columns (e.g. 1–2.1 mm IDs) would also be able to handle monoamines, amino acids and peptides simultaneously, but one should expect a decreased sensitivity compared to using cLC. Although a ZIC-cHILIC column was chosen for this platform, the more common ZIC-HILIC column should perform nearly as well, but with some differences in selectivity^31^, i.e. the ability to separate various compounds.

Simultaneous determination of both polar analytes as well as neuropeptides would be far more challenging with the more common reversed phase LC approach, which separates according to hydrophobicity (typically limiting applicably to compounds with intermediate/low polarity). In contrast, HILIC columns separate compounds based on a number of factors, e.g. partitioning between an immobilized water layer in the stationary phase and the mobile phase, normal phase/adsorption interactions, electrostatic interactions, hydrogen bonding but also reversed-phase (RP) interactions^18^,^32^,^33^,^34^. The use of HILIC may also enhance sensitivity compared to RP, as relatively high amounts of ACN are used in the HILIC mobile phase, which can increase the MS signal^35^.

Conventional cLC injections are limited to 5–10 μL injections. Here, we used on-line SPE to perform much larger injection volumes (100 μL) to lower the detection limits without compromising chromatography. On-line SPE also greatly reduces manual steps in sample preparation, which can be a central source of error in analysis^36^. On the other hand, on-line SPE-LC can be prone to clogging when handling complex samples^22^. To avoid this, the AFFL feature was employed, ensuring robust performance. AFFL-SPE-cLC is straightforward to install^17^,^22^ and is compatible with common commercial LC-MS instrumentation, only requiring one extra pump and one switching valve. Whole blood samples are notorious for clogging LC equipment. We largely attribute the absence of problems analysing whole blood samples to the AFFL feature, which captures particulate matter with a filter that is back-flushed after each injection.

Using a zwitter-ionic HILIC phase also for SPE was superior to using ion exchange (IEX) phases or porous graphitic carbon (PGC) phases; IEX-HILIC and PGC-HILIC were associated with reduced LC performance due to poor retention, or the ZIC-cHILIC separation column being incompatible with aqueous/very salty SPE loading solvents. This does not appear to be a significant issue when employing larger bore SPE-LC systems^37^, but becomes a factor with small-bore systems (such as cLC), where system void volumes can play a larger role in system performance^38^. The “HILIC-HILIC” combination for SPE-LC has also been successfully used in proteomics and glycoproteomics^38^,^39^. To the authors' knowledge, this is the first report on “HILIC-HILIC” SPE-LC for studying endogenous NTs/metabolites.

To ensure full analyte retention on the HILIC SPE, the aqueous standards were diluted with ACN/H_2_O (70/30). Based on this, ACN with 0.1% FA was used to precipitate proteins from the whole blood samples, in a 1 + 7.5 ratio (blood + precipitant). This allowed the blood samples to be directly injected onto the AFFL-SPE system, without any evaporation/re-dissolving step, making sample preparation both time-efficient and easy, with a minimum of manual work. Since whole blood could be analysed without plasmafication, additional time and manual work were saved, and the blood sample could be analysed less than one hour after the blood was collected.

When performing targeted determination and quantifications, triple quadrupole (3Q) instruments are often preferred, as they are known for their excellent quantitative capabilities. Orbitrap instruments are however superior^29^ in resolution, and more typically used for untargeted analysis (e.g. comprehensive proteomics). This study exemplifies that precise quantifications of small metabolites can be successful with an Orbitrap instrument. In fact, linear calibration curves were obtained even without correction with internal standards (Supplementary Fig. S4), which implies relatively trustworthy quantifications for untargeted metabolomics as well. Considering that miniaturized HILIC-Orbitrap-based LC-MS systems work well for both proteomics^38^,^39^ and NTs (and likely other metabolites) as described here, it is worth considering that a single instrument set-up similar to that described here can be used for both bottom-up proteomics and metabolomics, allowing their simultaneous determination.

Few methods on quantitative determination of NTs in human whole blood have been published, and reference materials are to the authors' knowledge, not available. However, the endogenous levels quantified by our method were comparable (but somewhat higher) to levels reported in plasma samples (Supplementary Table S2). The reason is likely the high uptake of several neurotransmitters in blood platelets^40^, and indeed the levels of some of the analytes were lower in plasma, especially serotonin (measured with a non-validated method).

We have demonstrated that AFFL-SPE-cLC-MS can be used for quantifying NTs in small amounts of blood, with minimal sample preparation only including ACN precipitation. The platform can be used for single-run chromatography of a wide range of NTs (monoamines, amino acids and peptides). Next steps will be to continue adding analytes to the platform, towards more comprehensive “neurotransmitter-omics”, and validating the quantification of e.g. oxytocin, whose labeled internal standard has become commercially available at the time of submission (Sigma Aldrich, St. Louis, MO, USA). Therapeutics affecting NT activity may also be monitored simultaneously, as HILIC is compatible with drug analysis^41^.

## Methods

For method development/validation/measurements, compound mixtures used were: an external standard mixture consisting of commercially obtained compounds (≥99% purity) (“analyte standard mix”, see Supplementary Table S3); a mixture of isotopically labelled analogs of the analytes, serving as internal standards (“internal standard mix”, see Supplementary Table S3).

### Study subjects and sample preparation

All subjects gave written informed consent, and the blood collection was approved by the Regional Ethics Committee (2011/1337/REK S-OE D). All methods were carried out in accordance with the approved guidelines and regulations.

Whole blood samples from veins were collected in vials containing K2 EDTA (BD, East Rutherford, NJ, USA), immediately aliquoted and stored at −80°C until the time of analysis. For validation studies, 8 blood samples were pooled prior to precipitation. In 1.5 mL polypropylene vials (Eppendorf, Hamburg, Germany), aliquots of 100 μL of thawed whole blood were mixed with 50 μL of an internal standard working solution mixture (made by diluting the internal standard mixture 50 times, see Supplementary Table S5 for details). Subsequently, 100 μL of ACN/H_2_O (70/30) was added (to ensure equal volumes compared to that of spiked samples in validation studies, see below) and finally 750 μL of cold ACN with 0.1% FA was rapidly added. The vials were vigorously vortexed for 20 seconds before kept at 4°C for 20 minutes, to maximize the protein precipitation. Finally, the vials were centrifuged for 10 minutes at 5000 rpm (Centrifuge 5415 R, Eppendorf). Approximately 700 μL of the supernatant were collected, and 100 μL of this were injected directly onto the AFFL-SPE-cLC-MS system.

### AFFL-SPE-cLC-MS System

For all chemicals and equipment used, see Supplementary Chemicals and equipment. An automated filtration/filter backflush (AFFL)-SPE-cLC-MS system^22^ was used in this study (see Supplementary Fig. S1 for an illustration of the entire system and Fig. 3 for details of the switching system). In position 1 (load) the auto sampler injects 100 μL of sample, and then the sample is pumped through a 1 μm stainless steel filter fitted in a union. The analytes will pass through the filter and be retained on the SPE column, while un-retained non-polar compounds and solvent will go directly to waste. The purpose of the filter is to prevent larger particles from the sample e.g. precipitates, to reach and clog the SPE column. In position 2 (inject) the 10 port valve is switched and the cLC pump elutes the analytes off the SPE column and onto the analytical column. Simultaneously, the SPE pump connected to the auto-sampler will back-flush the filter and wash off particles/precipitates. For pump 1 the loading mobile phase was 100% ACN, the flow rate was 75 μL/min and the loading time 2 minutes. For pump 2 the LC mobile phase consisted of 70% ACN and 30% 100 mM ammonium formate (pH 3, adjusted with 1% formic acid), and the flow rate was 4 μL/min. The column temperature was 30°C. After being separated on the analytical column the analytes will be transferred to the ESI where they are ionized before they enter the Orbitrap MS (See Supplementary Table S1 for all MS parameters).

### Method validation

The method was validated with samples made by spiking 100 μL of pooled in-house whole blood with 100 μL of the analyte standard mix at seven different concentration levels: XL, L, LM, M, HM, H and XH (Supplementary Table S4). The rest of the procedure was the same as for the sample preparation (see section above). The calibration samples were made in the same way as the validation samples, and all samples were analysed on the same day as they were prepared. The within-day repeatabilities were found by analysing 6 sample replicates of validation samples with L, M and H concentration levels on the same day, while validation samples with L, M and H concentrations were analysed on 5 different days to investigate the between-day repeatabilities. The linearity was examined with standard solutions (n = 3) and with validation samples (n = 7). The determination of the cLOD was limited by the lack of blood without NTs (blank matrix). However, a crude estimate was calculated by extrapolation (for details, see Supplementary note Calculations of cLOD).

The recovery was investigated in the following way: one pooled blood sample was spiked with NT analytes (XH level, to minimize contribution from endogenous levels) before protein precipitation, and another pooled blood sample was spiked with NT analytes after protein precipitation. To calculate the recovery, equation (1) was used. *A* is the peak area of the analyte and *A_is_* is the peak area of the internal standard, respectively.
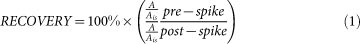
Stability was tested at room temperature, 4°C, −20°C and with thawing/freezing. Validation samples from the first validation day were re-analysed after 24 h, 48 h, 1 week and after one and two thaw/freeze cycles, and peak areas were compared with that of day 1.

### Quantification of neurotransmitters in whole blood

To quantify the amounts of NTs in the whole blood samples, calibration curves based on calibration samples with concentrations from XL to XH (Supplementary Table S4), constructed using Excel, were used. Since the calibration solutions were made by spiking blood which already contained the neurotransmitters of interest, the endogenous levels of NTs were calculated using the regression equation from the calibration curve, corrected for the endogenous level (for details, see Supplementary note Calculation of NT concentrations, and Supplementary Fig. S5).

## Supplementary Material

Supplementary Information Supplementary information

## Figures and Tables

**Table 1:** MS/MS transitions and retention times. The table presents the MS/MS transitions for all the NTs examined in this study, and for the internal standards of the six small NTs. Isotope-labelled internal standards for the neuropeptides were not available at the time the study was conducted. Retention times are also included

	Analyte		Internal standard		
Neurotransmitter	Precursor ion *(m/z)*	Product ion *(m/z)*	Precursor ion *(m/z)*	Product ion *(m/z)*	Retention time (min)
**GABA**	104.07	87.0443	106.09	89.0569	6.9
**PEA**	122.10	105.0699	127.13	110.1012	4.0
**Dopamine**	154.09	137.0593	158.11	141.0843	6.2
**Serotonin**	177.09	160.0751	181.13	164.1003	4.8
**Adrenaline**	184.10	166.0856	190.13	172.1233	6.7
**Tryptophan**	205.10	188.0699	208.12	191.0887	4.7
**Oxytocin**	1007.40	723.26	-	-	5.4
**Vasopressin**	1084.45	*	-	-	8.4

*No considerable fragmentation for vasopressin was observed with fragmentation energy of 25 or 35%, so the precursor ion *m/z* (1084.455) was used for identification.
