# Supplementary material for: Liquid chromatography-mass spectrometry platform for both small neurotransmitters and neuropeptides in blood, with automatic and robust solid phase extraction
Source: Sci Rep. 2015 Mar 20;5:9308. doi: 10.1038/srep09308 (PMC5380133; doi:10.1038/srep09308)
Supplement: Supplementary Information [file srep09308-s1.pdf]

Liquid chromatography-mass spectrometry platform for both small neurotransmitters and neuropeptides in blood, with automated and robust solid phase extraction

Elin Johnsen[1,*], Siri Leknes[2,3], Steven Ray Wilson[1,*] and Elsa Lundanes[1]

[1]Department of Chemistry, University of Oslo, PO Box 1033, Blindern, NO-0315, Oslo, Norway

[2]Department of Psychology, University of Oslo, PO Box 1094, Blindern, NO-0317, Oslo, Norway

[3]The intervention Centre, Oslo University Hospital, PO Box 4950, Nydalen, NO-0424 Oslo, Norway

*Corresponding authors

Chemicals and equipment

Mobile phases were made with HPLC grade acetonitrile (ACN) from VWR (West Chester, PA, USA), and HPLC water (Chromasolv Plus for HPLC), ammonium formate (NH_4HCO_2), formic acid (FA, 98 %), and ascorbic acid, all purchased from Sigma Aldrich (St. Louis, MO, USA). Tracy SPE columns (ZIC-HILIC, SCX, WCX, PGC, 0.5 (ID) x 5 mm) were obtained from G&T Septech (Kolbotn, Norway), while the ZIC-HILIC and the ZIC-cHILIC analytical columns (0.3 mm x 150 mm, 3.5 µm) were from Merck Millipore (Merck KGaA, Darmstadt, Germany). Two pumps were used: a Hitachi L-7110 (Merck) as SPE pump, and an Agilent 1100 series capillary gradient pump (Agilent Technologies, Palo Alto, CA, USA) with a max 20 µL/min flow cell, as LC pump. Injection was performed by a G1313 A ALS standard auto sampler (Agilent Technologies). A 10 port two-position switching valve (1/16", 0.25 mm bore) from Valco (Houston, TX, USA) controlled by the LC pump's Chemstation software (Agilent), performed the column switching. The AFFL filter was of stainless steel (1/16 µm, 1 µm screen) and fitted in a union (1/16 µm, 0.25 mm bore), both obtained from Valco. The column was connected to a Q-Exactive Orbitrap mass spectrometer (Thermo Scientific, Bellafonte, PA, USA) with a capillary electrospray ionization source (ESI, Thermo). The ESI was operated in positive ionization mode with a capillary voltage of 2500 V. Mass spectra were acquired in the *m/z* range 80-210. The transitions between the precursor ions to the most intensive fragment ions were monitored for qualitative determination of each NT in targeted MS/MS mode. XCalibur software was used for controlling the MS and for data collection.

Neurotransmitters and internal standards

Dopamine HCl, γ-aminobutyric acid (GABA), (-) epinephrine, (-), 2-phenylethylamine, serotonin HCl, and L-tryptophan were obtained from Sigma. 2-Phenyl-d_5-ethylamine, 4-aminobutyric-4,4-d_2 acid, 2-(3,4-dihydroxyphenyl)ethyl-1,1,2,2-d_4-amine HCl, epinephrine-2,5,6,α,β,β-d_6, *L*-tryptophan-2,3,3-d_3 and serotonin- α,α,β,β-d_4 creatinine sulphate complex, all purchased from CDN isotopes (Quebec, Canada) were used as internal standards for quantification. Stock solutions of all NT analytes and IS were prepared as 5 mM solutions in 50 % H_2O (HPLC grade) and 50 % 0.1 M HCl (Sigma) and stored at -80° C.

Figures

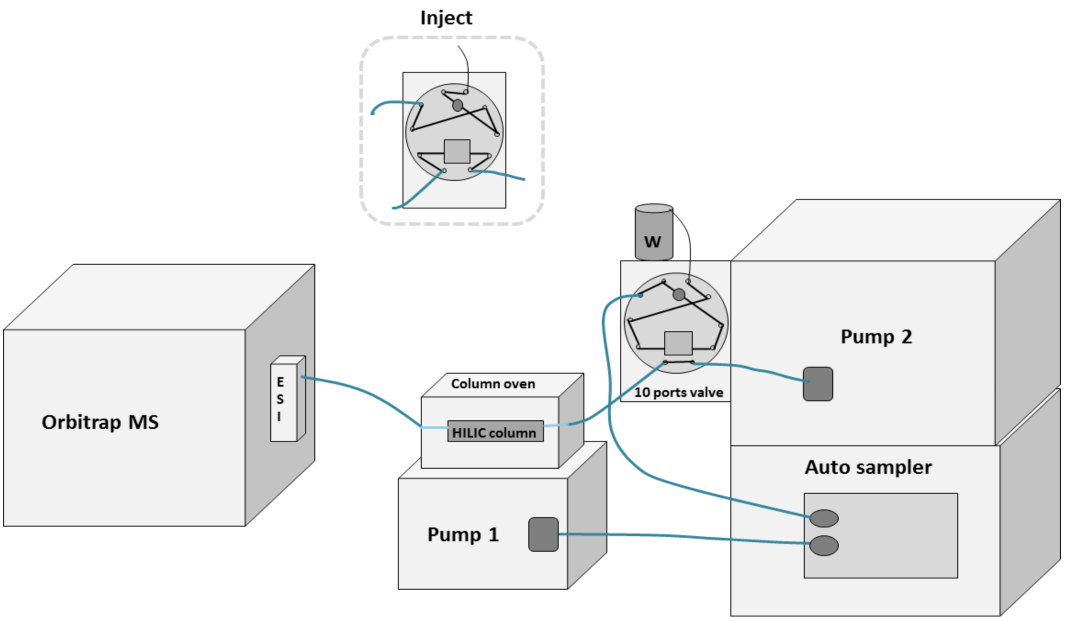

Supplementary Figure S1: **Illustration of the AFFL-SPE-cLC-MS platform.** Pump 1 is connected to the autosampler and transfers the injected sample to the 10 ports valve where the filter and the SPE column are located. When the valve is in position 1 (load) as illustrated here, the sample is loaded onto the SPE while the solvent goes directly to waste. When the valve is switched to position 2 (inject), showed in the figure above the system, pump 2 elutes the analytes off the SPE and onto the analytical column which is situated in a column oven set to 30° C. After being separated on the HILIC column the anlytes are transferred to the ESI where they are ionized before they enter the Orbitrap MS where they are detected by their *m/z* values and MS/MS fragmentations.

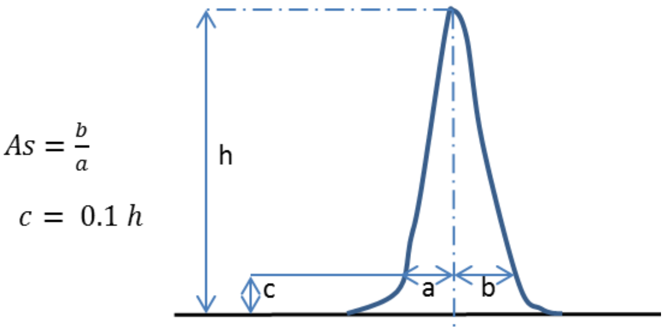

Supplementary Figure S2: Illustration of the method used for calculating the asymmetry factor (As).

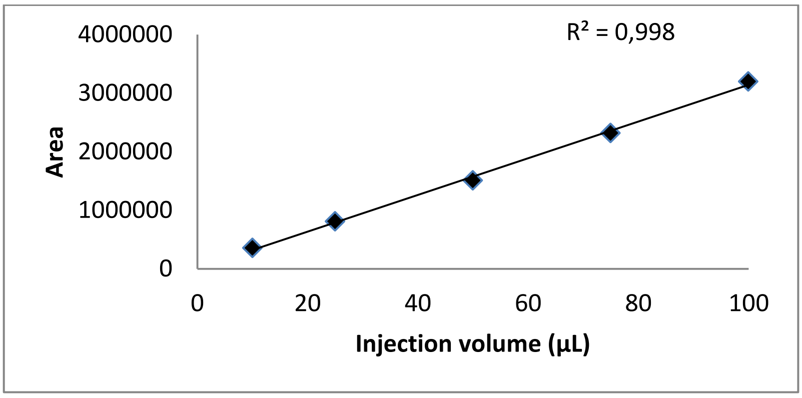

Supplementary Figure S3: Plot of peak area vs injection volume. The injection volume was investigated from 10 to 100 µL (max injection volume of the auto sampler), by plotting the peak areas against the injection volumes (n = 2 for each point). This gave a linear response with r^2 = 0.998, showing that no breakthrough occurred for these volumes.

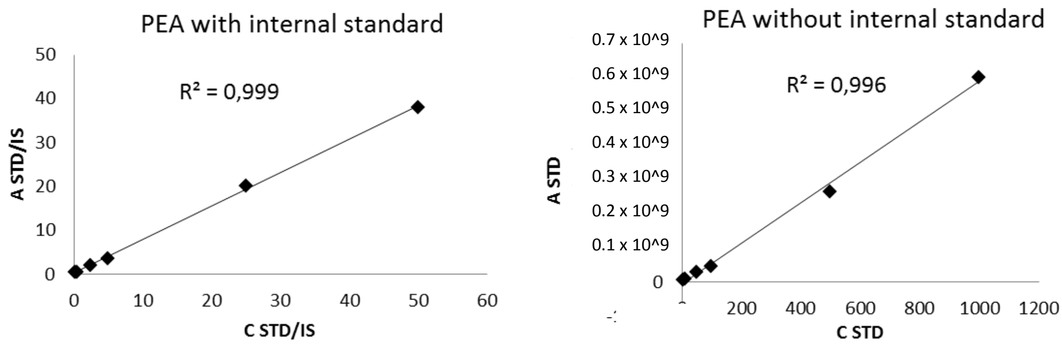

Supplementary Figure S4: Calibration curves with and without internal standard. To see if it was possible to obtain trustworthy quantifications without using internal standards, calibration curves were constructed based on only the NT concentrations and NT peak areas (n = 2 for each point). The R^2 value was still better than 0.99 for all NT analytes, when no internal standard was used, exemplified with calibration curves constructed for PEA *with* internal standard (left) and *without* internal standard (right).

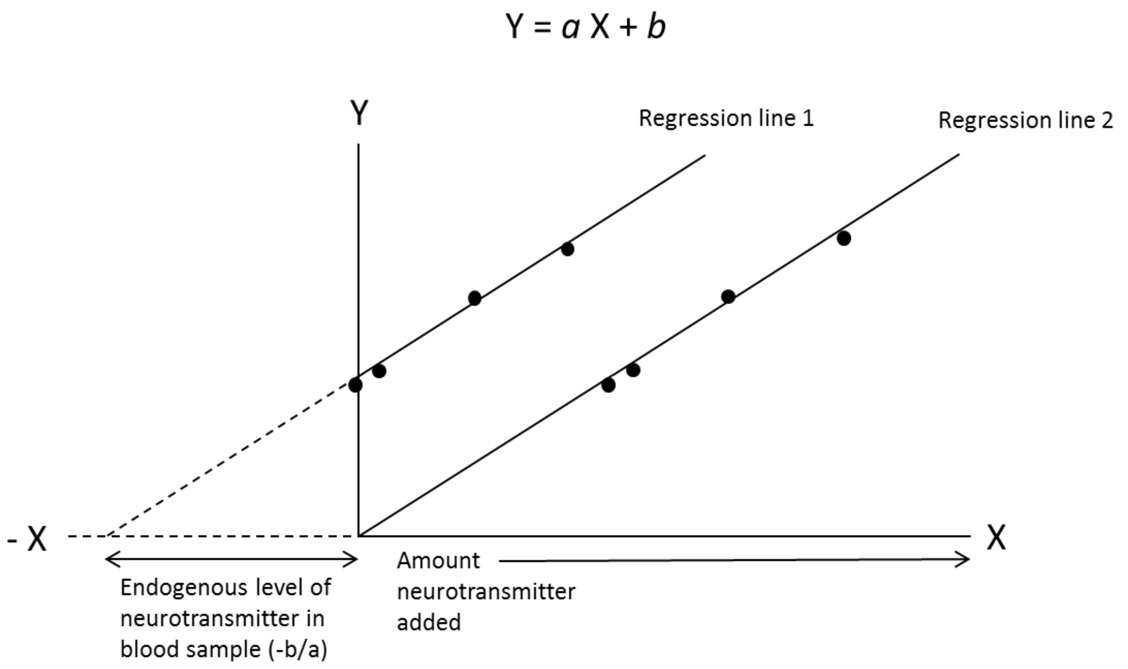

Supplementary Figure S5: Calculation of NT concentrations. Illustration of the method used for correcting for the endogenous levels of NTs in blood. Regression line 1 is the original line, and regression line 2 is the corrected one.

Tables

Supplementary Table S1: **MS parameters**.

**Parameters tune file**		**Parameters MS/MS file**	
Sheat gas flow rate	5	Resolution	70 000
Aux gas flow rate	0	AGC target	1e5
Sweep gas flow rate	0	Max injection time	200 ms
Spray voltage (kV)	2.5	Isolation width	1 m/z
Capillary temperature	240	Fragmentation energy (NCE)	25 %
S-lens RF level	50		
Aux gas heater temperature	0		

Supplementary Table S2: **NTs identified/measured in whole blood samples.** Six NTs were quantified in whole blood samples from two volunteers. The results were compared to results obtained in other studies. Some of the differences can possibly be explained by the fact that in the other studies all of the NTs were quantified in plasma, not whole blood (WB), except for serotonin which was quantified in both plasma and whole blood, but the values varied a lot from study to study.

	**Sample 1**	**Sample 2**	**Other studies**
**GABA**	109 nM	80 nM	122 nM[1]
**PEA**	13 nM	13 nM	931 pg/mL (~8 nM)[2], 1130 pg/mL (~9 nM)[3]
**Dopamine**	< 1 nM	< 1 nM	0.01-0.35 nM[4]
**Serotonin**	83 nM	76 nM	0.36 nM[5], 4.8 nM (713 nM in WB)[6]
**Adrenaline**	0.8 nM	0.4 nM	0.01-1.3 nM[4]
**Tryptophan**	4 µM	4 µM	~1 µg/mL (~5 µM)[7]

Supplementary Table S3: Concentrations of the individual NTs in the STD mix and the internal standard mix. For the method development stock solution mixtures (STD mix) were made once a month and stored at -20° C. The same was done for internal standards (IS mix). Fresh working solutions were made daily by diluting the stock solution mixtures with ACN/H_2O (70/30) to appropriate concentrations. For validation, new stock solution mixtures with all compounds were made. These mixtures were stored at -80° C, and diluted to appropriate concentrations same day as the analysis.

	**GABA**	**PEA**	**Dopamine**	**Serotonin**	**Adrenaline**	**Tryptophan**
**STD MIX (µM)**	50	50	0.5	50	0.5	2500
**IS MIX (µM)**	20	20	10	15	10	300

Supplementary Table S4: Concentration levels of NT standards used in validation and calibration samples. The six concentration levels of the NTs used in the validation and calibration samples given in nM. Since the expected levels of endogenous NTs varied from pM to µM, each concentration level (XL-XH) contained individual concentrations of the different NTs.

	**Concentrations levels (nM)**						
**Neurotransmitter**	XL	L	ML	M	HM	H	XH
**GABA**	5	10	50	100	500	1000	5000
**PEA**	5	10	50	100	500	1000	5000
**Dopamine**	0.05	0.1	0.5	1	5	10	50
**Serotonin**	5	10	50	100	500	1000	5000
**Adrenaline**	0.05	0.1	0.5	1	5	10	50
**Tryptophan**	250	500	2500	5000	25 000	50 000	250 000

Supplementary Table S5: Concentration levels of internal standards used in validation, calibration and real samples. A working solution with all internal standards were made by diluting the IS mix (see Table S3) 50 times. This solution was added to all validation and calibration samples, and also to the real blood samples. The concentration levels of internal standards were adjusted according to the expected levels of endogenous NTs, but also after their individual signal intensities, to ensure that all internal standards could be easily quantified. The table includes the final concentration of the internal standards in all the samples (validation/calibration/real), and also how many moles of internal standard which were injected.

**Internal standards**	Working solution MIX (nM)	Final concentration in all samples (nM)	Moles injected (pmol)
**GABA**	400	20	2
**PEA**	400	20	2
**Dopamine**	200	10	1
**Serotonin**	300	15	1.5
**Adrenaline**	200	10	1
**Tryptophan**	6000	300	30

Notes

Calculation of efficiency

Efficiency (N) was calculated using the formula:

$$N = 5.54 \times \left(\frac{t_R}{t_{w_{0.5}}}\right)^2$$

t_R is the retention time of the analyte, and $t_{W0.5}$ is the width at half of the peak height.

Mobile phases

70% ACN and 30 % 100 mM ammonium acetate: the more polar compounds (e.g. dopamine) were not eluted off the column. Poor peak shape were obtained for the other NTs.

70 % ACN and 30 % 50 mM ammonium formate: irreproducible results with severe band broadening and sometimes no elution of the more polar compounds. No significant decrease in background noise was observed.

75 % ACN and 25 % 120 mM ammonium formate: band broadening was observed for GABA and the efficiency went from 3400 (70 % ACN) to 2500, a 26 % decrease.

80% ACN and 20 % 150 mM ammonium formate: peaks were broad and irregular, so N could not be measured properly.

Online oxidation

Preliminary studies were done on an Esquire 3000+ Ion trap MS (Bruker Daltonics, Billerica, MA, USA). Adding HCl to the NT stock solutions and having an acidic pH in the mobile phase were sufficient to prevent NT oxidation when this instrument was used. The method was moved to an Orbitrap MS when the need for better resolution and sensitivity arise, but severe oxidation (80-90%) of the catecholamines (dopamine and adrenaline) was then observed. When the flow was increased from 4 µL/min to 8 µL/min the oxidation decreased significantly, and it was assumed that the oxidation happened online during the analysis. It was also assumed that the ESI source was involved in the oxidation, since the configuration of this was quite different between the Orbitrap and ion trap MS. In the Orbitrap MS the voltage is on the emitter, and to protect the operator and the upstream equipment from being exposed to high voltage, a grounded contact is often placed upstream of the emitter electrode. But then a second upstream circuit is added and electrochemical reactions can occur[8]. In most cases, and for most analytes, this is not a problem, but when the analytes are easily oxidized (like the catecholamines), the mobile phase has a high conductivity (e.g. high amounts of salt) and the flow rate is relatively low, electrochemical reactions can occur. To avoid oxidation, 300 µM ascorbic acid was added to the mobile phase to act as an antioxidant [9]. No more oxidation was observed, and there were no interferences from the ascorbic acid even though it had the same *m/z* value as serotonin (*m/z* = 177). Since ascorbic acid is light sensitive, the mobile phase bottles were covered with alumina foil, and new mobile phases were made daily during the method validation and sample analyses.

SPE column materials

Porous graphitic carbon (Hypercarb): None of the NTs were retained.

Strong cation exchange (SCX): All NTs were retained, but to avoid severe band broadening, 30 % water had to be used in the loading mobile phase. Hence a water plug was eluted off the

SPE and onto the analytical column and this resulted in a loss of separation. And excessive amount of interferences from blood were also retained by the SCX material and subsequently released to the analytical column.

Weak cation exchange (WCX): All NTs were retained, but the same problem occurred as with the SCX. The recovery was also slightly lower.

Calculation of cLOD

The determination of the concentration limit of detection (cLOD) was limited by the lack of blood without NTs (blank matrix). Using standard samples instead would give an irrelevant cLOD since the matrix effects in the blood would not be accounted for. A crude estimate was therefore calculated for each NT by measuring the noise in the baseline close to the analyte peak in the MS/MS chromatograms from a pooled blood sample. Then the calibration curves from the validation were used to estimate the expected concentration at a signal to noise ratio of 3 (s/n = 3) in blood.

Calculation of NT concentrations

Peaks were manually integrated using the XCalibur software, and neurotransmitter concentrations were calculated with Excel using the formula "y = a x + b", equalling:

$$\frac{C_{NT}}{C_{IS}} = a \times \frac{A_{NT}}{A_{IS}} + b\,\frac{CNT}{CIS}$$

C_{NT} is the concentration of the NT of interest, C_{IS} is the concentration of the IS, A_{NT} is the area of the NT peak, while A_{IS} is the area of the peak corresponding to the IS. *a* is the slope of the regression line and *b* is the intercept with the y-axis of the calibration curve. To correct for the endogenous concentrations of NTs in the calibration samples, *C NT/C IS* was set to 0, and then A_{NT}/A_{IS} would correspond to *–b/a*. By adding this values (*-b/a*) to all X values, the regression line was moved on the positive side of the plot and the new regression equation, with *b* ~ 0, was used for calculating the concentrations of NTs in blood samples.

1 Bjork, J. M. *et al.* Plasma GABA levels correlate with aggressiveness in relatives of patients with unipolar depressive disorder. *Psychiatry Res.* **101**, 131-136 (2001).
2 Zhou, G., Miura, Y., Shoji, H., Yamada, S. & Matsuishi, T. Platelet monoamine oxidase B and plasma β-phenylethylamine in Parkinson's disease. *J. Neurol., Neurosurg. Psychiatry* **70**, 229-231 (2001).
3 Kawamura, K. *et al.* Improved method for determination of β-phenylethylamine in human plasma by solid-phase extraction and high-performance liquid chromatography with fluorescence detection. *J. Liq. Chromatogr. Related Technol.* **23**, 1981-1993 (2000).
4 Peaston, R. T. & Weinkove, C. Measurement of catecholamines and their metabolites. *Ann. Clin. Biochem.* **41**, 17-38 (2004).
5 Anderson, G. M., Hertzig, M. E. & McBride, P. Brief report: platelet-poor plasma serotonin in autism. *J. Autism Dev. Disord.* **42**, 1510-1514 (2012).
6 Hara, K., Hirowatari, Y., Shimura, Y. & Takahashi, H. Serotonin levels in platelet-poor plasma and whole blood in people with type 2 diabetes with chronic kidney disease. *Diabetes research and clinical practice* **94**, 167-171 (2011).
7 Bjork, J. M., Dougherty, D. M., Moeller, F. G. & Swann, A. C. Differential behavioral effects of plasma tryptophan depletion and loading in aggressive and nonaggressive men. *Neuropsychopharmacology* **22**, 357-369 (2000).
8 Kertesz, G. J. V. B. & Vilmos. Using the electrochemistry of the electrospray ion source. *Anal. Chem.* **79**, 5510-5520 (2007).
9 Plattner, S., Erb, R., Chervet, J.-P. & Oberacher, H. Ascorbic acid for homogenous redox buffering in electrospray ionization–mass spectrometry. *Anal. Bioanal. Chem.* **404**, 1571-1579 (2012).
